# Addendum: A FRET biosensor for necroptosis uncovers two different modes of the release of DAMPs

**DOI:** 10.1038/s41467-019-09536-9

**Published:** 2019-04-25

**Authors:** Shin Murai, Yoshifumi Yamaguchi, Yoshitaka Shirasaki, Mai Yamagishi, Ryodai Shindo, Joanne M. Hildebrand, Ryosuke Miura, Osamu Nakabayashi, Mamoru Totsuka, Taichiro Tomida, Satomi Adachi-Akahane, Sotaro Uemura, John Silke, Hideo Yagita, Masayuki Miura, Hiroyasu Nakano

**Affiliations:** 1 Department of Biochemistry, Toho University School of Medicine, 5-21-16 Omori-Nishi, Ota-ku, Tokyo, 143-8540 Japan; 2 Hibernation Metabolism, Physiology, and Development Group, Environmental Biology Division, Institute of Low Temperature Science, Hokkaido University, Kita 19, Nishi 8, Kita-ku, Sapporo, Hokkaido 060-0819 Japan; 3 Precursory Research for Embryonic Science and Technology, Japan Science and Technology Agency, Chiyoda-ku, Tokyo, 102-0075 Japan; 4 Department of Biological Sciences, Graduate School of Science, The University of Tokyo, 7-3-1 Bunkyo-ku, Tokyo, 113-0033 Japan; 5 Division of Cell Signaling and Cell Death, The Walter and Eliza Hall Institute of Medical Research, Parkville, VIC 3052 Australia; 6 Department of Medical Biology, University of Melbourne, Parkville, VIC 3050 Australia; 7 Laboratory of Molecular Biology and Immunology, Department of Biological Science and Technology, Faculty of Industrial Science and Technology, Tokyo University of Science, 6-3-1 Niijuku, Katsushika-ku, Tokyo, 125-8585 Japan; 8 Department of Food Science and Technology, Faculty of Applied Life Science, Nippon Veterinary and Life Science University, 1-7-1 Kyonancho, Musashino-shi, Tokyo, 180-8602 Japan; 9 Department of Physiology, Toho University School of Medicine, 5-21-16 Omori-Nishi, Ota-ku, Tokyo, 143-8540 Japan; 10 Department of Immunology, Juntendo University Graduate School of Medicine, 2-1-1 Hongo, Bunkyo-ku, Tokyo, 113-8421 Japan; 11 Department of Genetics, Graduate School of Pharmaceutical Sciences, The University of Tokyo, 7-3-1 Bunkyo-ku, Tokyo, 113-0033 Japan; 12 Host Defense Research Center, Toho University School of Medicine, 5-21-16 Omori-Nishi, Ota-ku, Tokyo, 143-8540 Japan

**Keywords:** Protein-protein interaction networks, Necroptosis, Cell death and immune response

## Abstract

The cDNA sequence of human SMART described in this Article was misreported, as described in the accompanying Addendum. This error does not affect the results or any conclusion of the Article.

Addendum to: *Nature Communications* 10.1038/s41467-018-06985-6, published online 26 October 2018

It has come to our attention that the human SMART biosensor reported in this Article does not contain SAGG repeats replacing the a and b regions corresponding to MLKL, as described. Following requests for murine and human SMART cDNAs, we were alerted that the sequence of human SMART is identical to human alpha15 (α15), which was a prototype of human SMART. We checked the sequence of the FRET biosensor integrated into HT29- and HaCaT cells, described in Figures 6 and 7, and found that it is human α15 and not our reported human SMART sequence (Figure. 1). To confirm that the human α15 construct behaves in the same way as the sensor described as human SMART, we regenerated stably transfected HT29 and HaCaT cells expressing the human α15 construct. As shown in Figure. 2, TNF+BV6+zVAD and PolyIC+BV6+zVAD induce an increase in the FRET/CFP ratio of human α15 in HT29 and HaCaT cells, respectively. This error does not affect the results or any conclusions of the Article.

**Figure 1:**
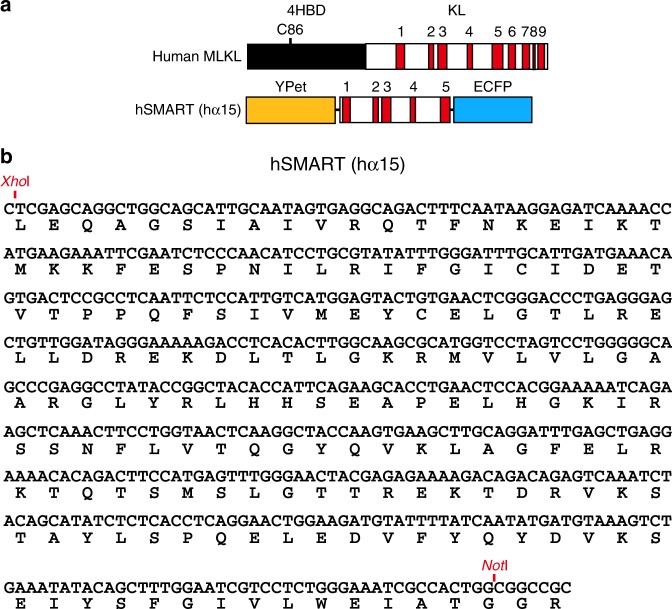
Fig. 1

**Figure 2:**
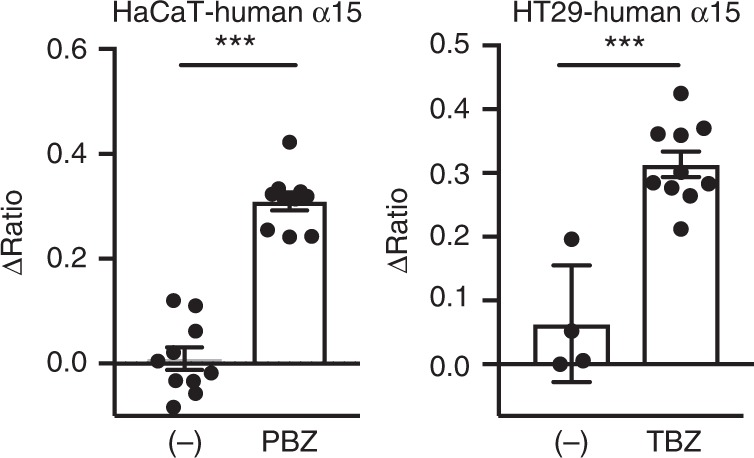
Fig. 2

