# Supplementary material for: A FRET biosensor for necroptosis uncovers two different modes of the release of DAMPs
Source: Nat Commun. 2018 Oct 26;9:4457. doi: 10.1038/s41467-018-06985-6 (PMC6203740; doi:10.1038/s41467-018-06985-6)
Supplement: Supplementary file 1 — Supplementary Information [file 41467_2018_6985_MOESM1_ESM.pdf]

Supplementary Information.

A FRET biosensor for necroptosis uncovers two different modes of the release of DAMPs

Shin Murai[1], Yoshifumi Yamaguchi[2], Yoshitaka Shirasaki[3,4], Mai Yamagishi[4], Ryodai Shindo[1], Joanne M. Hildebrand[5,6], Ryosuke Miura[1,7], Osamu Nakabayashi[1], Mamoru Totsuka[8], Taichiro Tomida[9], Satomi Adachi-Akahane[9], Sotaro Uemura[4], John Silke[5,6], Hideo Yagita[10], Masayuki Miura[11], Hiroyasu Nakano[1,12]*

Supplementary Figures 1 to 13.

Supplementary Table 1.

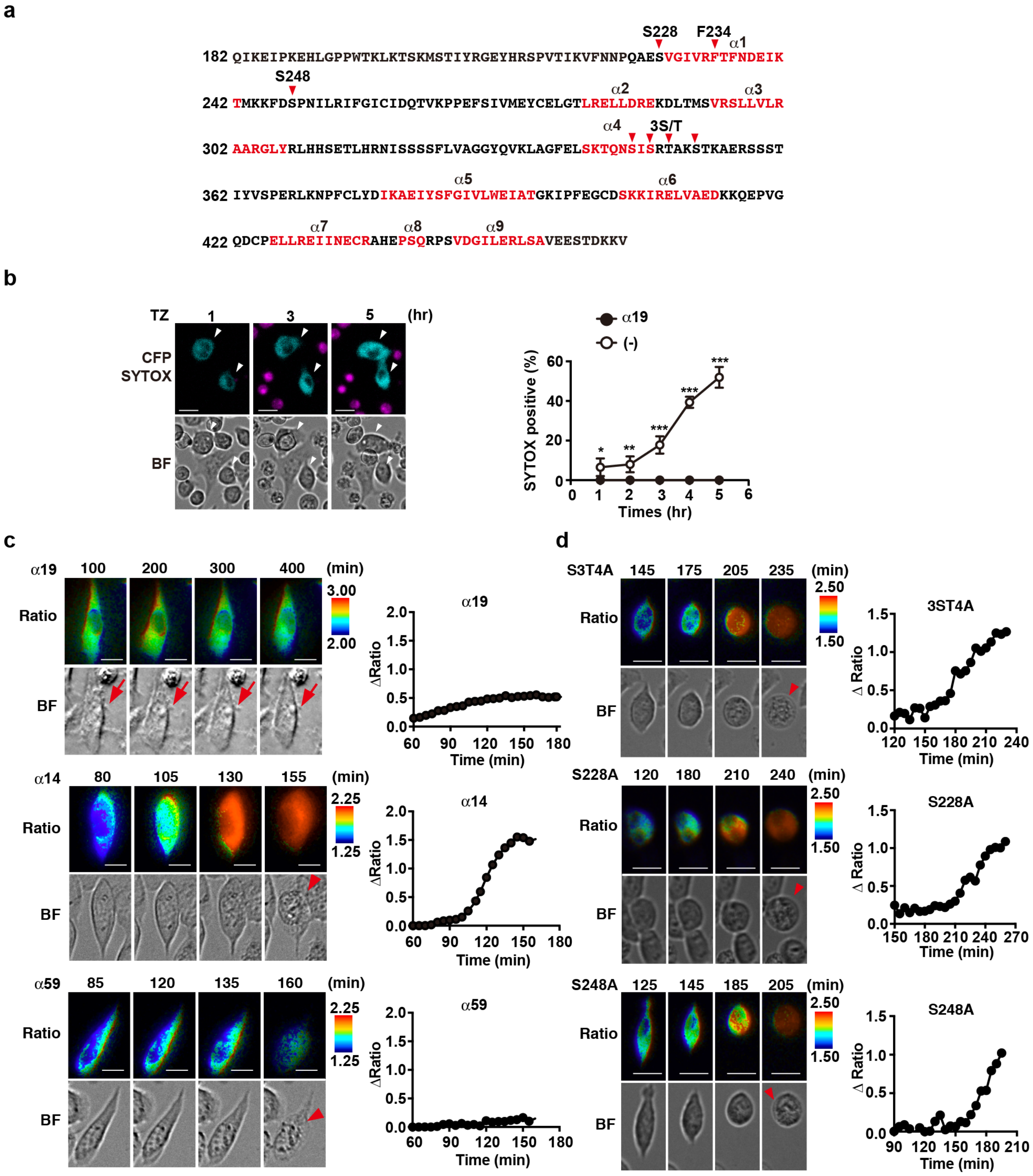

Supplementary Figure 1 | α14 monitors TNF + zVAD-induced necroptosis of L929 cells.

(**a**) Amino acid sequence of the KL domain of murine MLKL and the α helices. Red arrowheads indicate potential phosphorylation sites. F234 indicates critical phenylalanine for binding to RIPK3. Amino acids forming the α helices are highlighted in red letters. (**b**) L929

cells were transiently transfected with α19 and then stimulated with TZ for the indicated times. Representative images of L929 cells at the indicated times after stimulation (left). CFP + SYTOX and BF indicate merged images of CFP (cyan) and SYTOX Orange (magenta), and bright field, respectively. White arrowheads indicate CFP (α19)-positive live cells. Kinetics of percentages of SYTOX-positive dead cells among CFP-positive (+) or negative (-) cells after TZ stimulation (right). Numbers of SYTOX-positive cells were estimated by counting cells in several high-power fields at the indicated times. Results are mean ± s.d. Statistical significance was determined using the unpaired two-tailed Student's *t* test. **P*<0.05; ***P*<0.01; ****P*<0.001. (**c, d**) α14 monitors necroptosis in living cells and mutation of serine resides does not affect the FRET induction. L929 cells were transiently transfected with the indicated vectors, and then treated with TZ. The FRET/CFP ratio was calculated as in Fig. 1(b). Representative images of the ratio (left) and kinetics of ΔFRET/CFP ratio (right) of a single cell expressing the indicated biosensors (n=10 to 20 cells). Ratio and BF indicate the FRET/CFP ratio and bright field images, respectively. Red arrows and arrowheads indicate living cells expressing α19 and cells undergoing membrane rupture, respectively. Scale bars, 20 μm. Colour scales indicate pseudocolour images of the FRET/CFP ratio. All results are representative of two independent experiments.

a

Δα2α3

```
CAGGCCGAAAGTGTTGGAATCGTACGGTTCACTTTCAATGACGAGATCAAAACCATGAAG
Q A E S V G I V R F T F N D E I K T M K
KpnI
AAATTCGATTCTCCCAACATCTTGCGTATATTTGGATCCAGTGCTGGTGGTAGTGCTGGT
K F D S P N I L R I F G S S A G G S A G

GGTAGTGCTGGTGGTAGTGCTGGTGGTAGTGCTGGTGGTTCCGGCAGTGCTGGTGGTAGT
G S A G G S A G G S A G G S G S A G G S

GCTGGTGGTAGTACCAGTGCTGGTGGTAGTGCTGGTGGTAGTGCTGGTGGTAGTGCTGGT
A G G S T S A G G S A G G S A G G S A G

GGTAGTGCTGGTGGTTCCGGCAGTGCTGGTGGTAGTGCTGGTGGTAGTACCAGTGCTGGT
G S A G G S G S A G G S A G G S T S A G

GGTAGTGCTGGTGGTAGTGCTGGTGGTAGTGCTGGTGGTAGTGCTGGTGGTTCCGGCAGT
G S A G G S A G G S A G G S A G G S G S

GCTGGTGGTAGTGCTGGTGGTAGTACCAGTGCTGGTGGTAGTGCTGGTGGTAGTGCTGGT
A G G S A G G S T S A G G S A G G S A G
BspE1
GGTAGTGCTGGTGGTAGTGCTGGTGGTTCCGGATTTGAGTTAAGCAAAACACAGAATTCC
G S A G G S A G G S G F E L S K T Q N S

ATCAGCCGGACAGCAAAGAGC
I S R T A K S
```

Δα3

```
XhoI
CTCGAGCAAGCCGAATCCGTTGGGATTGTACGGTTTACCTTCAACGACGAGATCAAGACG
L E Q A E S V G I V R F T F N D E I K T

ATGAAGAAGTTTGACAGCCCCAACATTCTGCGCATTTTCGGGATCTGCATAGACCAGACA
M K K F D S P N I L R I F G I C I D Q T

GTGAAGCCTCCAGAGTTCTCTATCGTCATGGAGTACTGTGAACTGGGCACATTGAGGGAA
V K P P E F S I V M E Y C E L G T L R E

CTTCTGGATCGAGAGAAGGATCTGACTATGAGTGCAGGCGGCTCTGCTGGTGGGAGCGCG
L L D R E K D L T M S A G G S A G G S A

GGTGGATCAGCTGGAGGGTCTGCAGGCGGGAGTGCCGGAGGCTCAGCCGGTGGATCCGCC
G G S A G G S A G G S A G G S A G G S A
HindIII
GGTGGAAGCGCTGGCGGCGGATATCAGGTGAAGCTT
G G S A G G G Y Q V K L
```

b

α14(+)

```
XhoI
CTCGAGCAGGCCGAAAGTGTTGGAATCGTACGGTTCACTTTCAATGACGAGATCAAAACC
L E Q A E S V G I V R F T F N D E I K T

ATGAAGAAATTCGATTCTCCCAACATCTTGCGTATATTTGGGATTTGCATTGATCAAACA
M K K F D S P N I L R I F G I C I D Q T

GTGAAGCCCCCTGAGTTCTCCATTGTCATGGAGTACTGTGAACTTGGAACCCTGAGGGAG
V K P P E F S I V M E Y C E L G T L R E
SacI                                FspI
CTCCTGGATAGAGAAAAAGACCTCACAATGAGTGTGCGCAGCCTCCTAGTCCTGAGGGCA
L L D R E K D L T M S V R S L L V L R A

GCCAGAGGCTTATACAGGCTACACCATTCGGAAACACTCCACAGAAACATCAGCAGCTCC
A R G L Y R L H H S E T L H R N I S S S
HindIII
AGTTTCCTCGTAGCCGGAGGCTACCAAGTAAAGCTT
S F L V A G G Y Q V K L
```

Δabc

```
XhoI
CTCGAGCAGGCCGAAAGTGTTGGAATCGTACGGTTCACTTTCAATGACGAGATCAAAACC
L E Q A E S V G I V R F T F N D E I K T

TCAGCTGGAGGCAGTGCTGGCGGCAGCGCAGGTGGAAGTGCGGGTGGCAGTGATCAAGCA
S A G G S A G G S A G G S A G G S D Q A

GGGGGGTCCGCTGGGGGCTCCGCTGGCGGGTCGGCCGGTGGAAGTGCAGGCCTGAGGGAG
G G S A G G S A G G S A G G S A G L R E
SacI                                FspI
CTCCTGGATAGAGCAGGAGGCAGCGCAGGTGGTGTGCGCAGCCTCCTAGTCCTGAGGGCA
L L D R A G G S A G G V R S L L V L R A

GCCAGAGGCTTATACGGTGGAAGCGCTGGTGGATCAGCCGGCGGAAGCGCTGGCGGCTCC
A R G L Y G G S A G G S A G G S A G G S
HindIII
GCTGGAGGATCAGCCGGAGGCTCCGCAGGAAAGCTT
A G G S A G G S A G K L
```

Δab (mSMART)

```
XhoI
CTCGAGCAGGCCGAAAGTGTTGGAATCGTACGGTTCACTTTCAATGACGAGATCAAAACC
L E Q A E S V G I V R F T F N D E I K T

TCAGCTGGAGGCAGTGCTGGCGGCAGCGCAGGTGGAAGTGCGGGTGGCAGTGATCAAGCA
S A G G S A G G S A G G S A G G S D Q A

GGGGGGTCCGCTGGGGGCTCCGCTGGCGGGTCGGCCGGTGGAAGTGCAGGCCTGAGGGAG
G G S A G G S A G G S A G G S A G L R E
SacI                                FspI
CTCCTGGATAGAGCAGGAGGCAGCGCAGGTGGTGTGCGCAGCCTCCTAGTCCTGAGGGCA
L L D R A G G S A G G V R S L L V L R A

GCCAGAGGCTTATACAGGCTACACCATTCGGAAACACTCCACAGAAACATCAGCAGCTCC
A R G L Y YRL H H S E T L H R N I S S S
HindIII
AGTTTCCTCGTAGCCGGAGGCTACCAAGTAAAGCTT
S F L V A G G Y Q V K L
```

Δa

```
XhoI
CTCGAGCAGGCCGAAAGTGTTGGAATCGTACGGTTCACTTTCAATGACGAGATCAAAACC
L E Q A E S V G I V R F T F N D E I K T

TCAGCTGGAGGCAGTGCTGGCGGCAGCGCAGGTGGAAGTGCGGGTGGCAGTGATCAAGCA
S A G G S A G G S A G G S A G G S D Q A

GGGGGGTCCGCTGGGGGCTCCGCTGGCGGGTCGGCCGGTGGAAGTGCAGGCCTGAGGGAG
G G S A G G S A G G S A G G S A G L R E
SacI                                FspI
CTCCTGGATAGAGAAAAAGACCTCACAATGAGTGTGCGCAGCCTCCTAGTCCTGAGGGCA
L L D R R S L L V L R V R S L L V L R A

GCCAGAGGCTTATACAGGCTACACCATTCGGAAACACTCCACAGAAACATCAGCAGCTCC
A R G L Y YRL H H S E T L H R N I S S S
HindIII
AGTTTCCTCGTAGCCGGAGGCTACCAAGTAAAGCTT
S F L V A G G Y Q V K L
```

c

hSMART

```
XhoI
CTCGAGCAGGCTGGCAGCATTGCAATAGTGAGGCAGACTTTCAATAAGGAGATCAAAACC
L E Q A G S I A I V R Q T F N K E I K T

ATGAAGAAATTCGGTACCAGTGCTGGTGGTAGTGCTGGTGGTAGTGCTGGTGGTAGTGCT
M K K F G T S A G G S A G G S A G G S A

GGTGGTAGTGCTGGTGGTTCCGGCAGTGCTGGTGGTAGTGCTGGTGGTACCCTGAGGGAG
G G S A G G S G S A G G S A G G T L R E

CTGTTGGATAGGGAAAGTGCTGGTGGTCTTGGCAAGCGCATGGTCCTAGTCCTGGGGGCA
L L D R E S A G G L G K R M V L V L G A
StuI
GCCCGAGGCCTATACCGGCTACACCAT...
A R G L Y R L H H...
```

Supplementary Figure 2 | Sequences of FRET biosensors. Nucleotide sequences and deduced amino acids of murine α14 mutants (**a, b**) and human SMART (**c**) are shown.

Replaced linker sequences are indicated by red characters. Restriction enzymes and their recognition sites are shown.

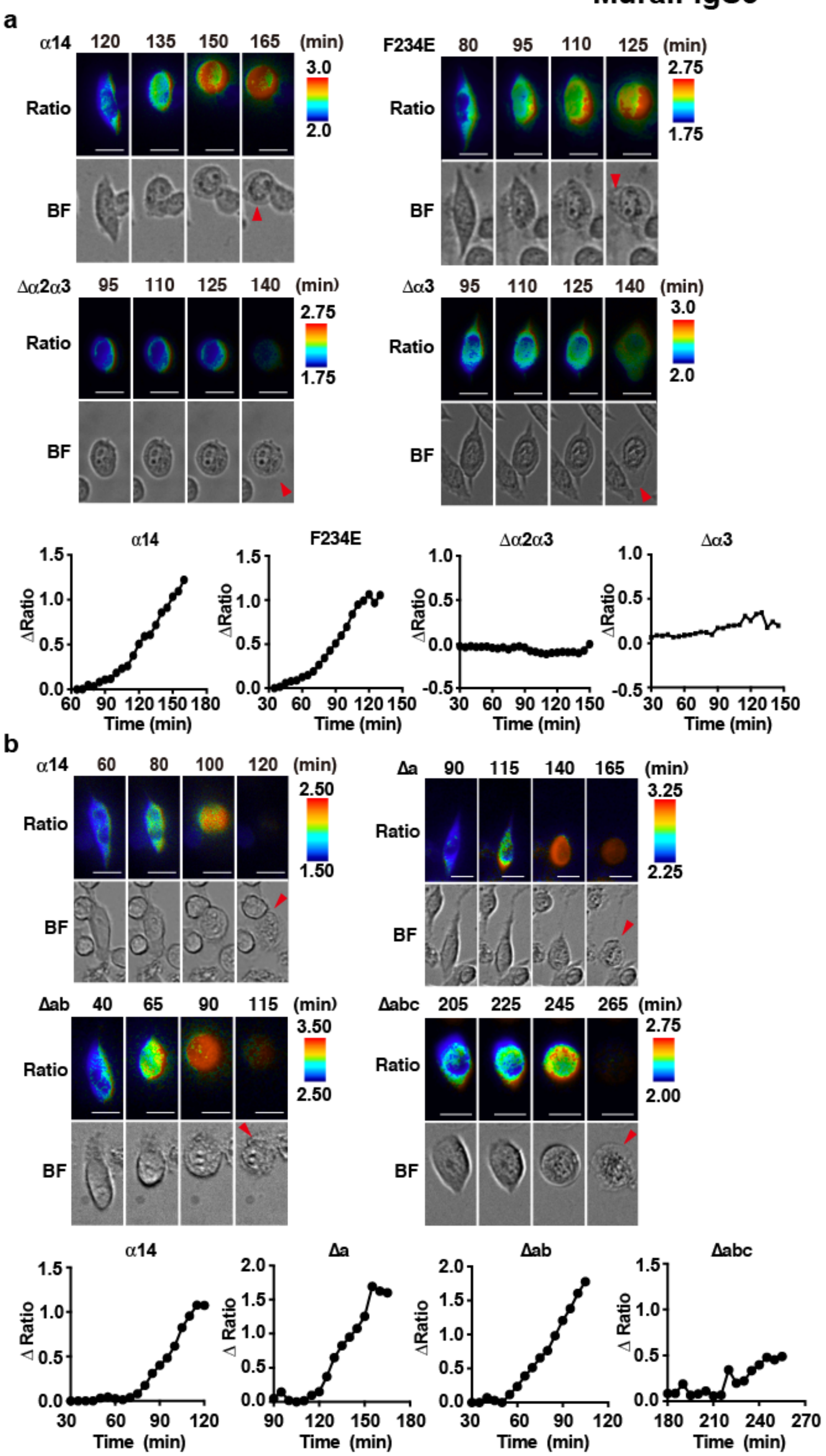

Supplementary Figure 3 | Interaction of α14 with RIPK3 is required for detection of

necroptosis. (**a, b**) L929 cells were transiently transfected with the indicated vectors, and then treated with TZ. The FRET/CFP ratio was analyzed as in Fig. 1(**b**). Representative images of the ratio (left) and kinetics of ΔFRET/CFP ratio (right) of a single cell expressing the indicated biosensors (n= at least 10 cells). Ratio and BF indicate the FRET/CFP ratio and bright field images, respectively. Red arrowheads indicate cells undergoing membrane rupture. Scale bars, 20 μm. Colour scales indicate pseudocolour images of the FRET/CFP ratio. All results are representative of two independent experiments.

MuraiFigS4

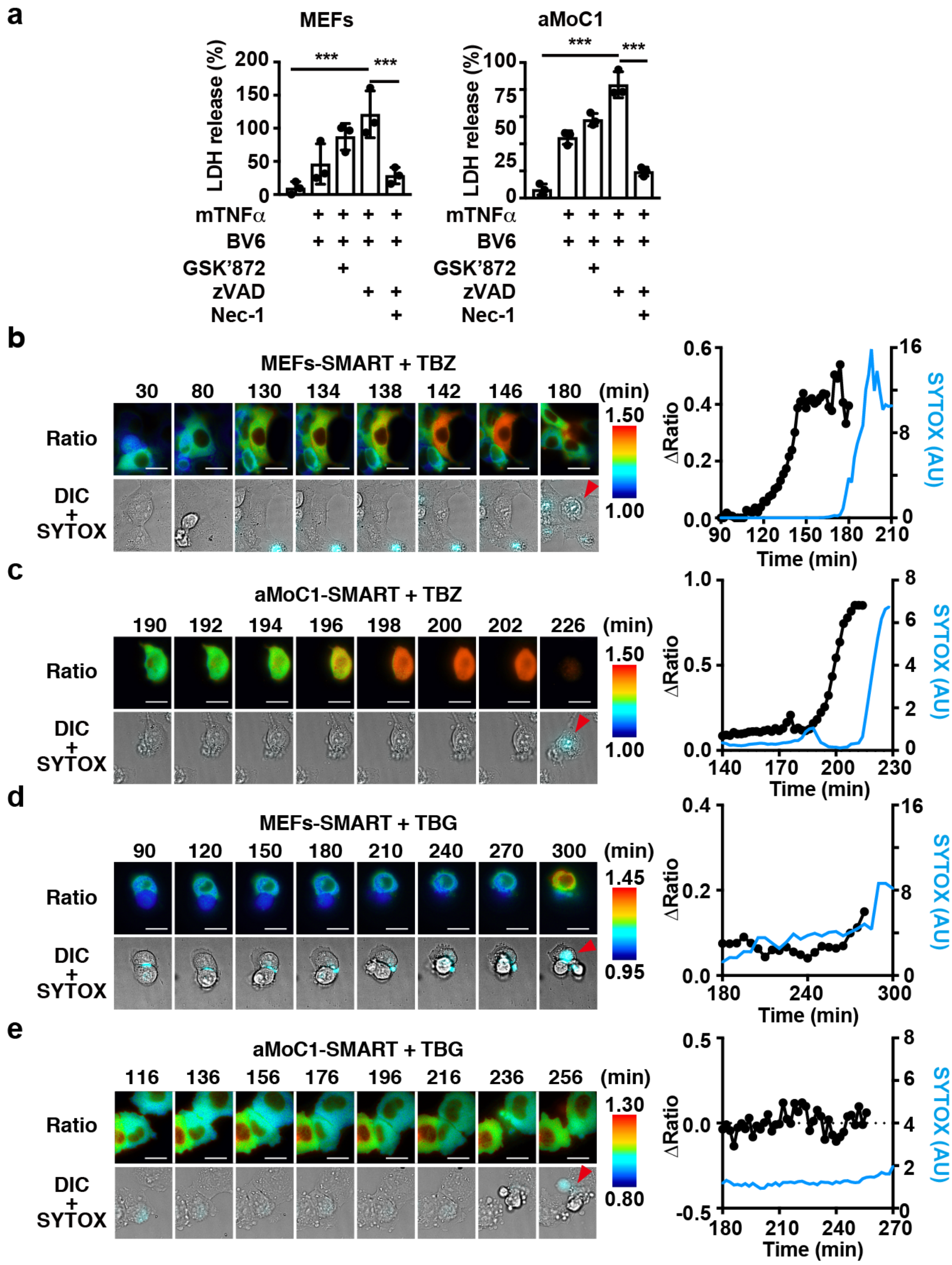

Supplementary Figure 4 | SMART monitors necroptosis in MEFs and aMoC1 cells. (a) MEFs and aMoC1 cells were stimulated with the combination of the indicated agents for 8 hours. Cell viability was determined by LDH release assay. Results are mean ± s.d of

triplicate samples. Statistical significance was determined using the one-way ANOVA test. ***$p<0.001$. (**b-e**) MEFs (**b, d**) and aMoC1 (**c, e**) cells stably expressing SMART, were stimulated with TNF/BV6/zVAD (TBZ) (**b, c**) or TNF/BV6/GSK'872 (TBG) (**d, e**). The FRET/CFP ratio was analyzed as in Fig. 1(**b**). Representative images of the ratio (left) and kinetics of ΔFRET/CFP (right) of a single cell expressing SMART (n=10-20 cells). Relative intensities of SYTOX Orange in the nucleus were calculated at the indicated times and are expressed as arbitrary units (AU). Ratio and DIC + SYTOX indicate the FRET/CFP ratio and merged images of DIC and SYTOX Orange, respectively. Red arrowheads indicate SYTOX-positive cells. Scale bars, 20 μm. Colour scales indicate pseudocolour images of the FRET/CFP ratio. All results are representative of two to three independent experiments.

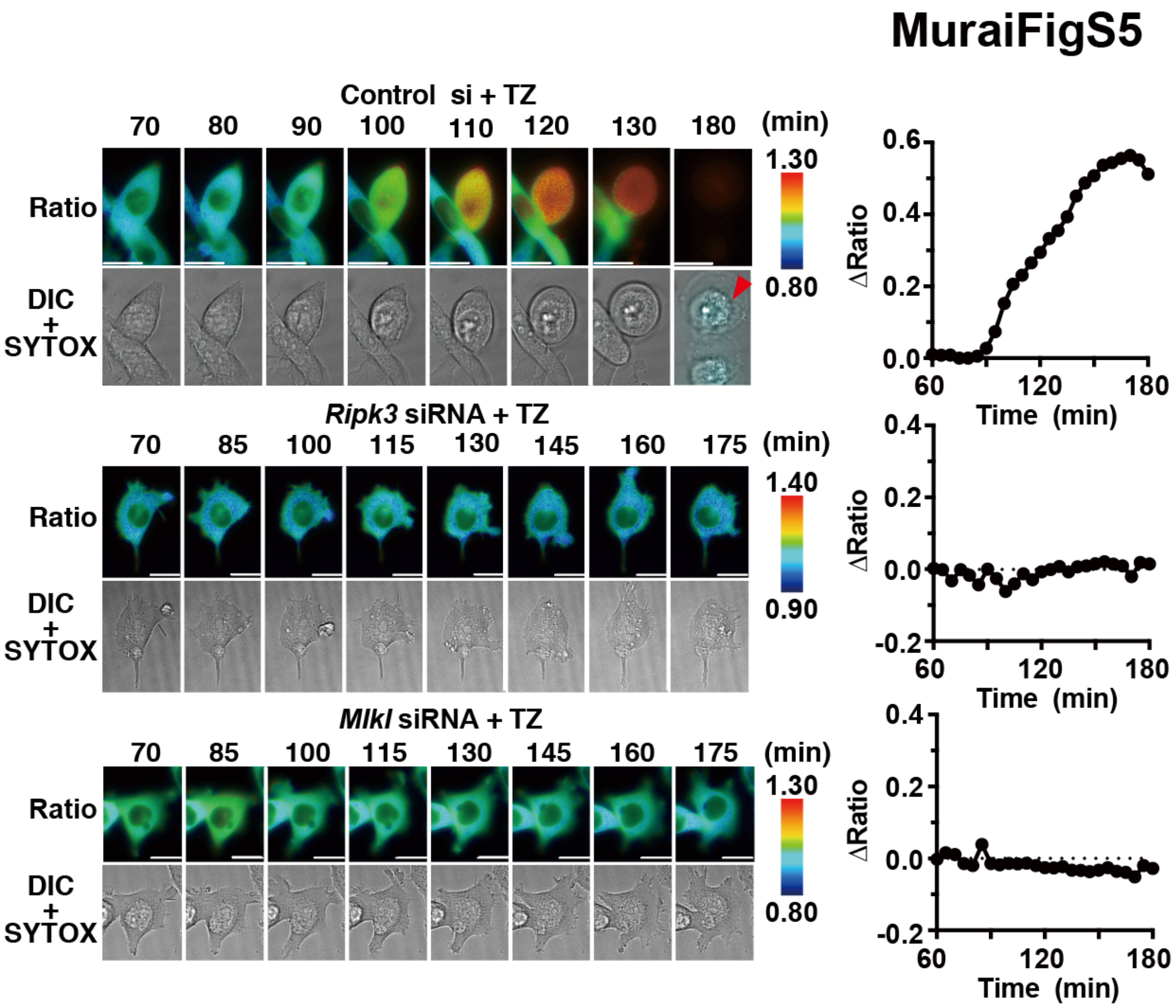

Supplementary Figure 5 | The increase in the FRET/CFP ratio of SMART depends on endogenous RIPK3 and MLKL. L929-SMART cells were transfected with control, *Ripk3,* or *Mlkl* siRNAs. After transfection, cells were stimulated with TZ. The FRET/CFP ratio was analyzed as in Fig. 1(**b**). Representative images of the ratio (left) and kinetics of ΔFRET/CFP (right) of a single cell expressing SMART (n=10-20 cells). Ratio and DIC + SYTOX indicate the FRET/CFP ratio and merged images of DIC and SYTOX Orange, respectively. Red

arrowheads indicate SYTOX-positive cells. Scale bars, 20 μm. Colour scales indicate pseudocolour images of the FRET/CFP ratio. Results are representative of three independent experiments.

MuraiFigS6

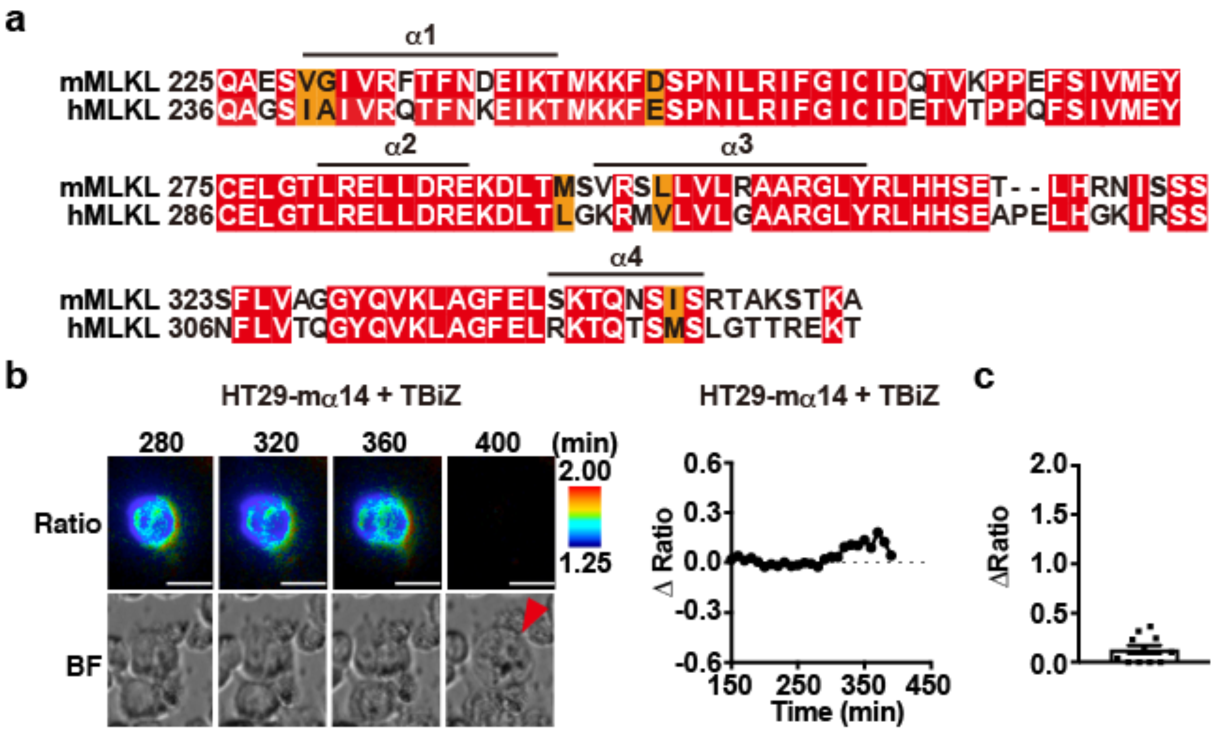

Supplementary Figure 6 | Murine α14 cannot monitor necroptosis of HT29 cells. (**a**) Alignment of amino acids of murine and human MLKL corresponding to the α1 to α4 helices. Amino acids in red and orange box indicate identical and homologous amino acids, respectively. The α1 to α4 helices are shown. (**b, c**) HT29 cells were transiently transfected with mα14 and then stimulated with human TNF/birinapant (an IAP inhibitor)/zVAD (TBiZ). The FRET/CFP ratio was analyzed as in Fig. 1(**b**). Representative images of ratio (left) and kinetics of ΔFRET/CFP (right) of a single cell (n=10-12 cells) (**c**). Ratio and BF indicate the FRET/CFP ratio and bright field images, respectively. Red arrowheads indicate cells undergoing membrane rupture. Scale bars, 20 μm. Maximum changes of ΔFRET/CFP ratio (**c**). Each dot indicates individual cell (n=11 cells). Colour

scales indicate pseudocolour images of the FRET/CFP ratio. Results are representative of two independent experiments. Error bars indicate s.e.m.

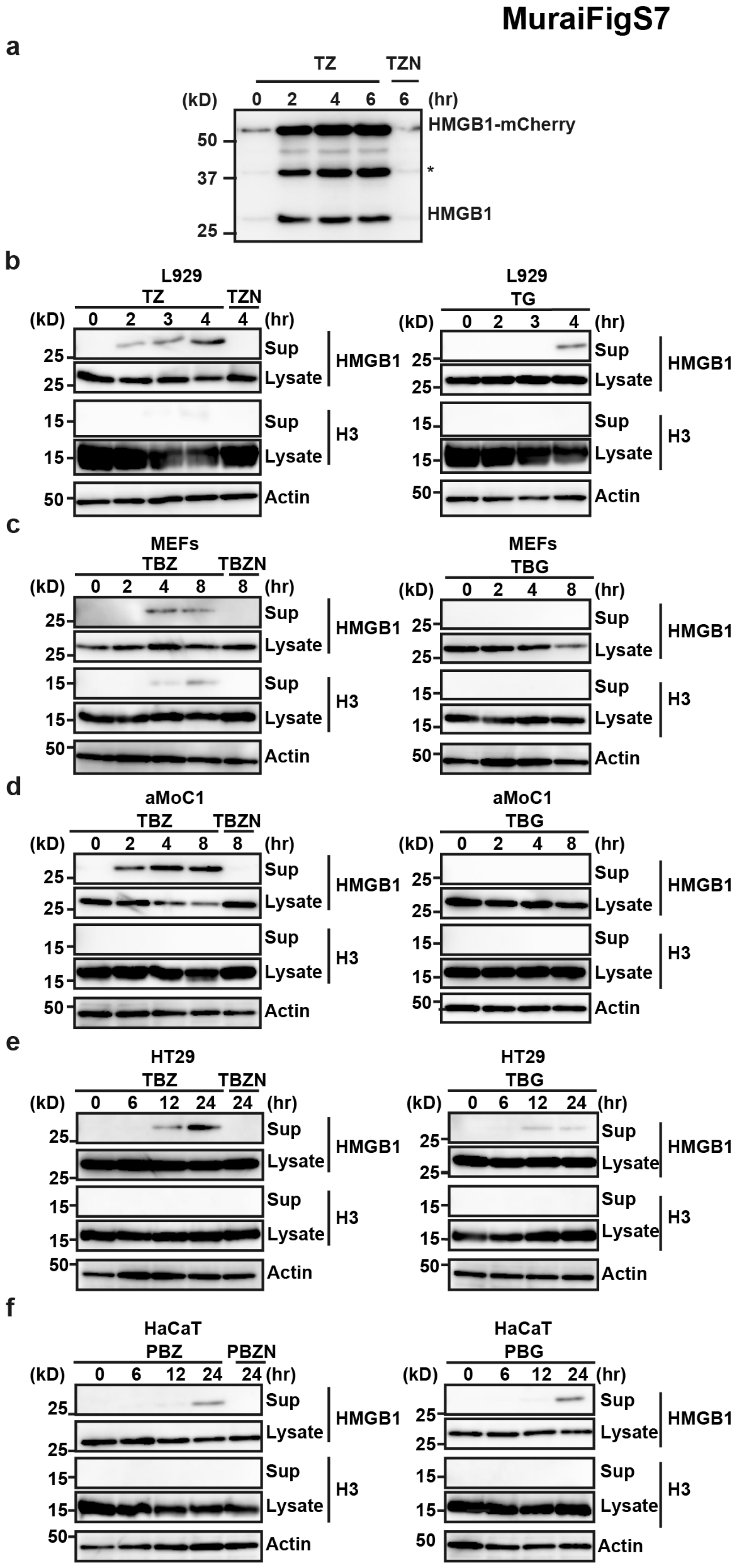

Supplementary Figure 7 | HMGB1 is released from cells undergoing necroptosis.

(**a**) L929 stably expressing HMGB1-mCherry cells were stimulated with TNF/zVAD (TZ) or TNF/zVAD/Nec-1 (TZN) for the indicated times, and released HMGB1 in the culture supernatant was analyzed by immunoblotting with anti-HMGB1 antibody. Asterisk indicates a truncated form of HMGB1-mCherry. (**b**) L929 cells were stimulated with TZ, TZN, or TNF/GSK'872 (TG) for the indicated times, and released HMGB1 and histone H3 in the culture supernatant (Sup) or whole cell lysates (lysate) were analyzed by immunoblotting with the indicated antibodies. (**c-e**) MEFs (**c**), aMoC1 (**d**), and HT29 (**e**) were stimulated with TNF/BV6/zVAD (TBZ), TNF/BV6/zVAD/Nec-1 (TBZN), or TNF/BV6/GSK'872 (TBG) and analyzed as in (**b**). (**f**) HaCaT cells were stimulated with Poly(I:C)/BV6/zVAD (PBZ), Poly(I:C)/BV6/zVAD/Nec-1 (PBZN), or Poly(I:C)/BV6/GSK'872 (PBG) and analyzed as (**b**). All results are representative of at least two independent experiments.

MuraiFigS8

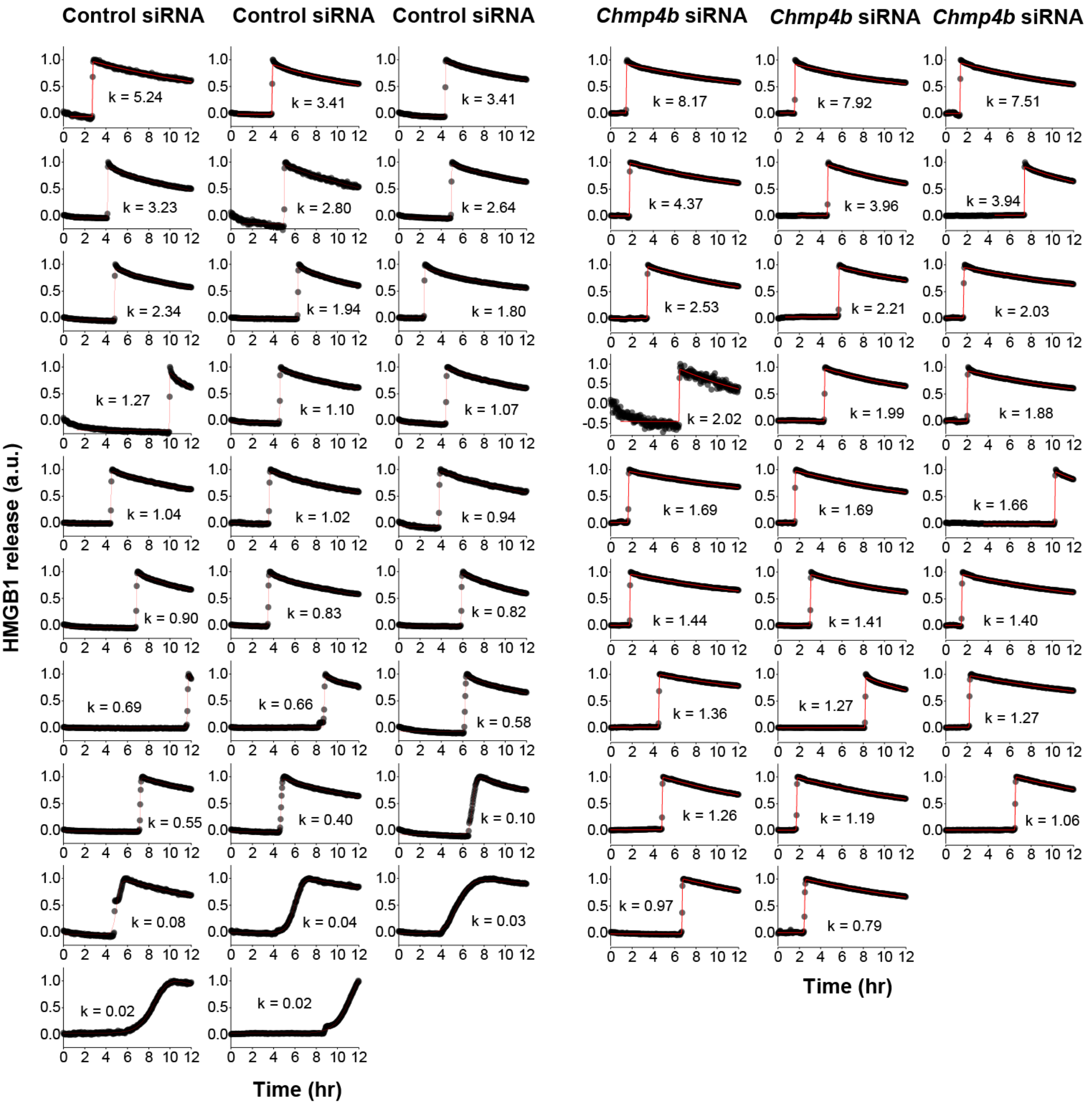

Supplementary Figure 8 | Knockdown of *Chmp4b* abolishes a sustained mode release of HMGB1. L929-SMART/HMGB1-mCherry cells were treated with control or *Chmp4b* siRNAs, and extracellular release of HMGB1-mCherry were analyzed as in Fig. 10 (**b**). Time indicates after TZ stimulation. Kinetics of the release of HMGB1 of a single cell is shown and steepness (*k*) is indicated.

MuraiFigS9

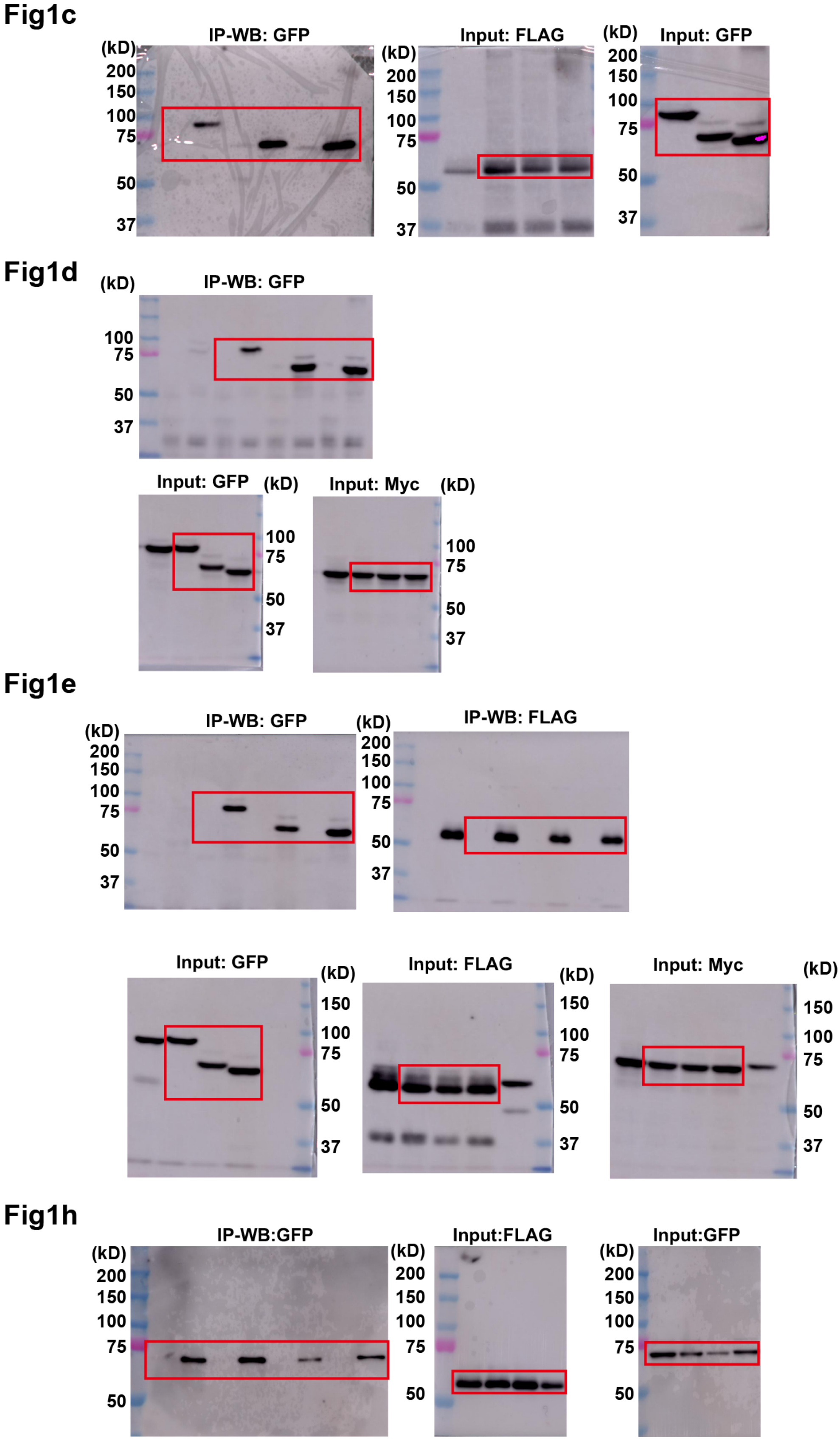

Supplementary Figure 9 | Uncropped blots for Fig. 1.

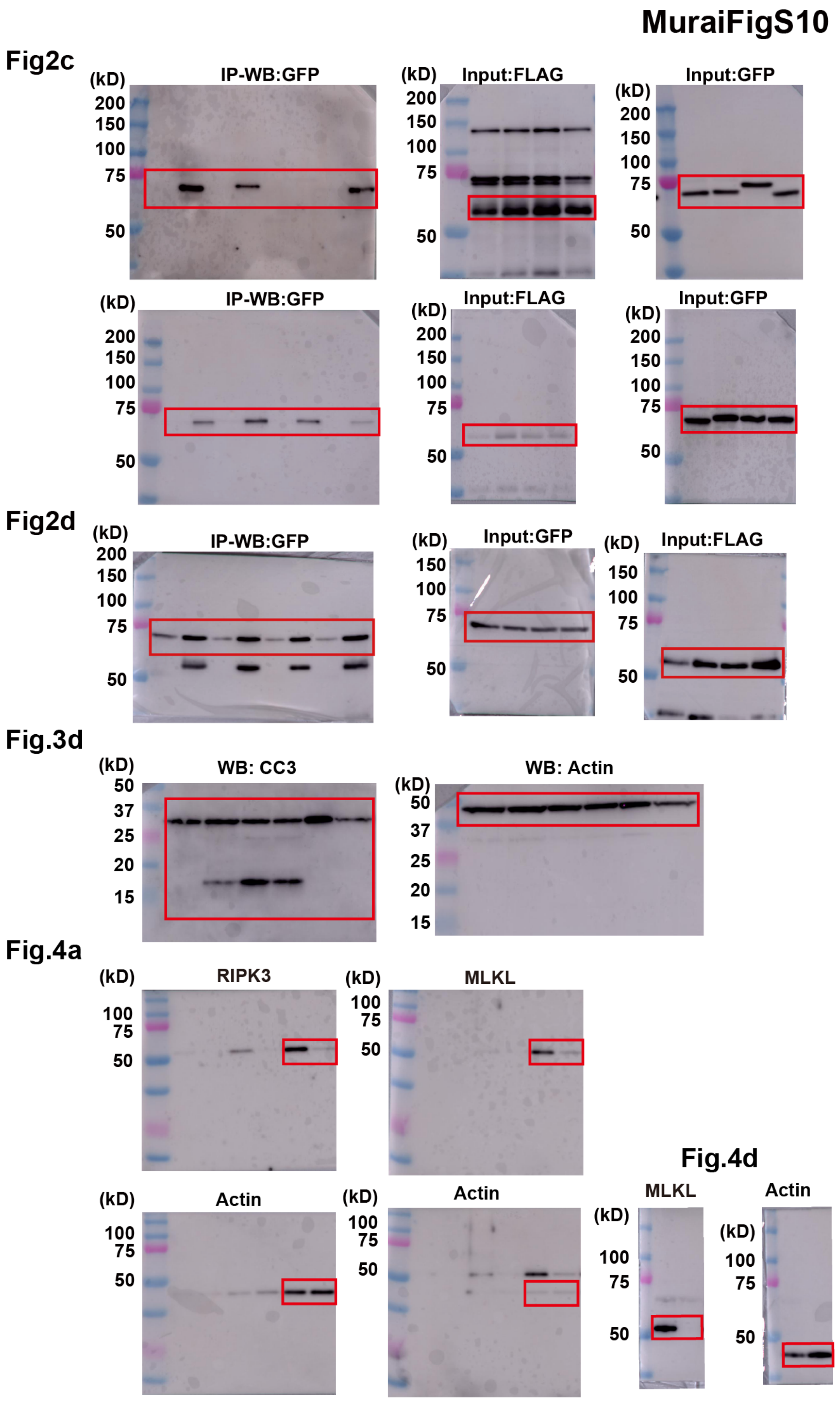

Supplementary Figure 10 | Uncropped blots for Figs. 2, 3, and 4.

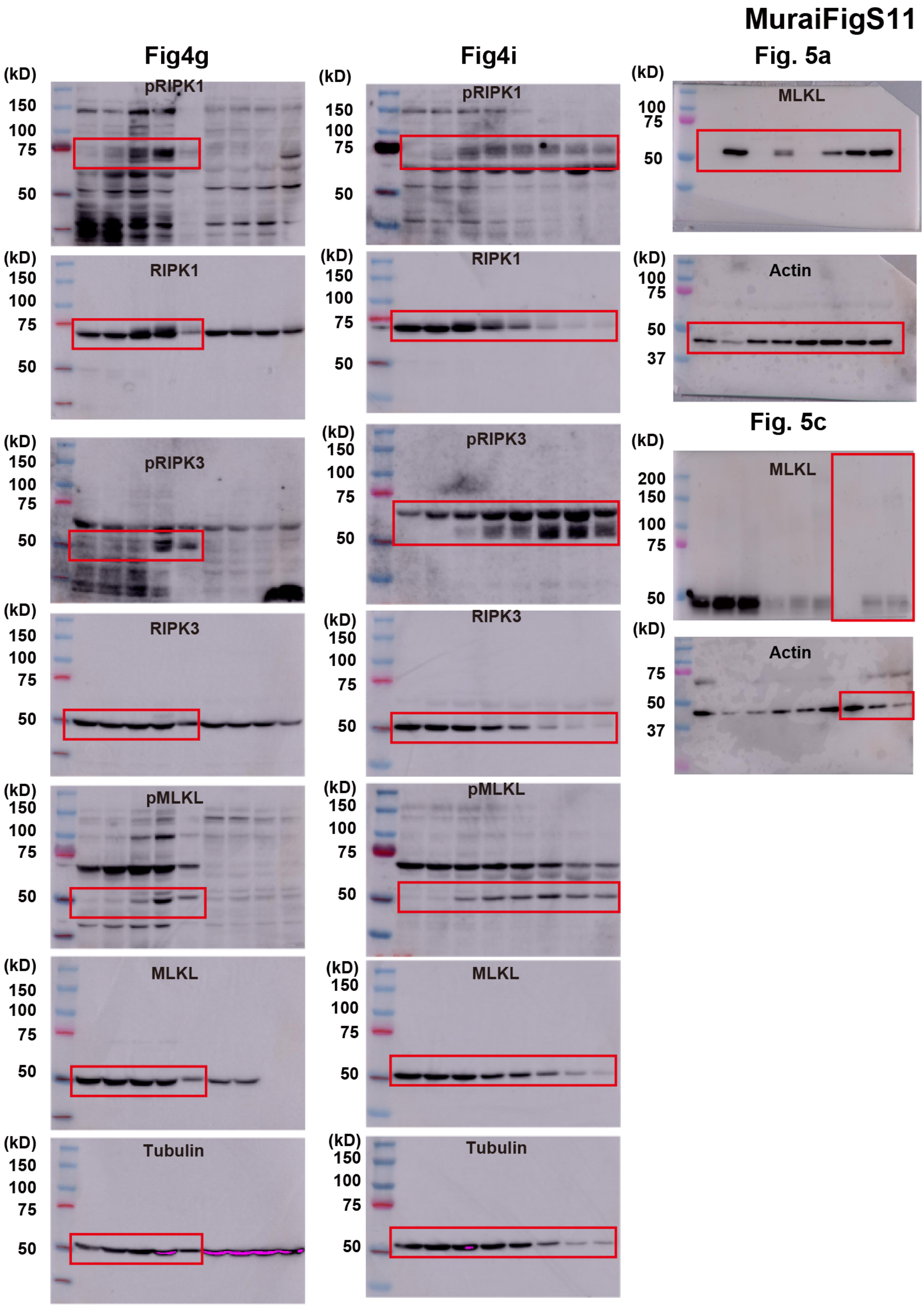

Supplementary Figure 11 | Uncropped blots for Figs. 4 and 5.

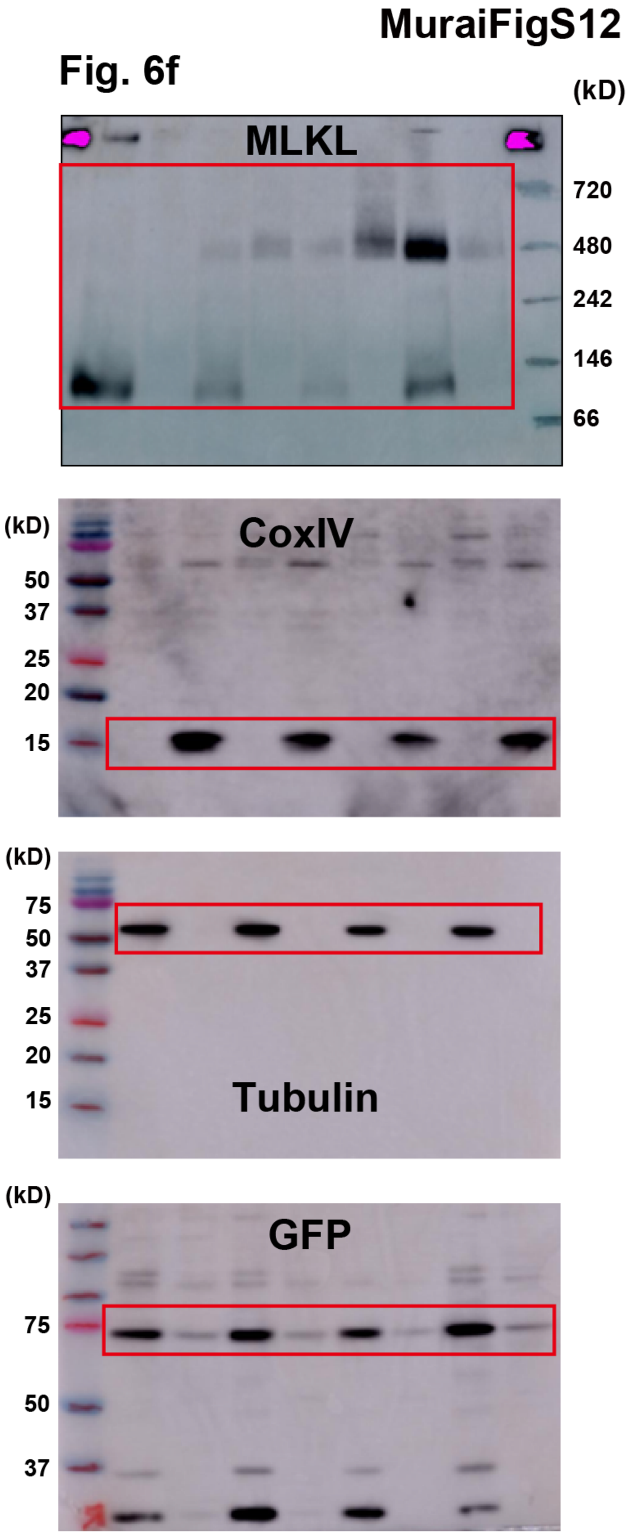

Supplementary Figure 12 | Uncropped blots for Fig 6.

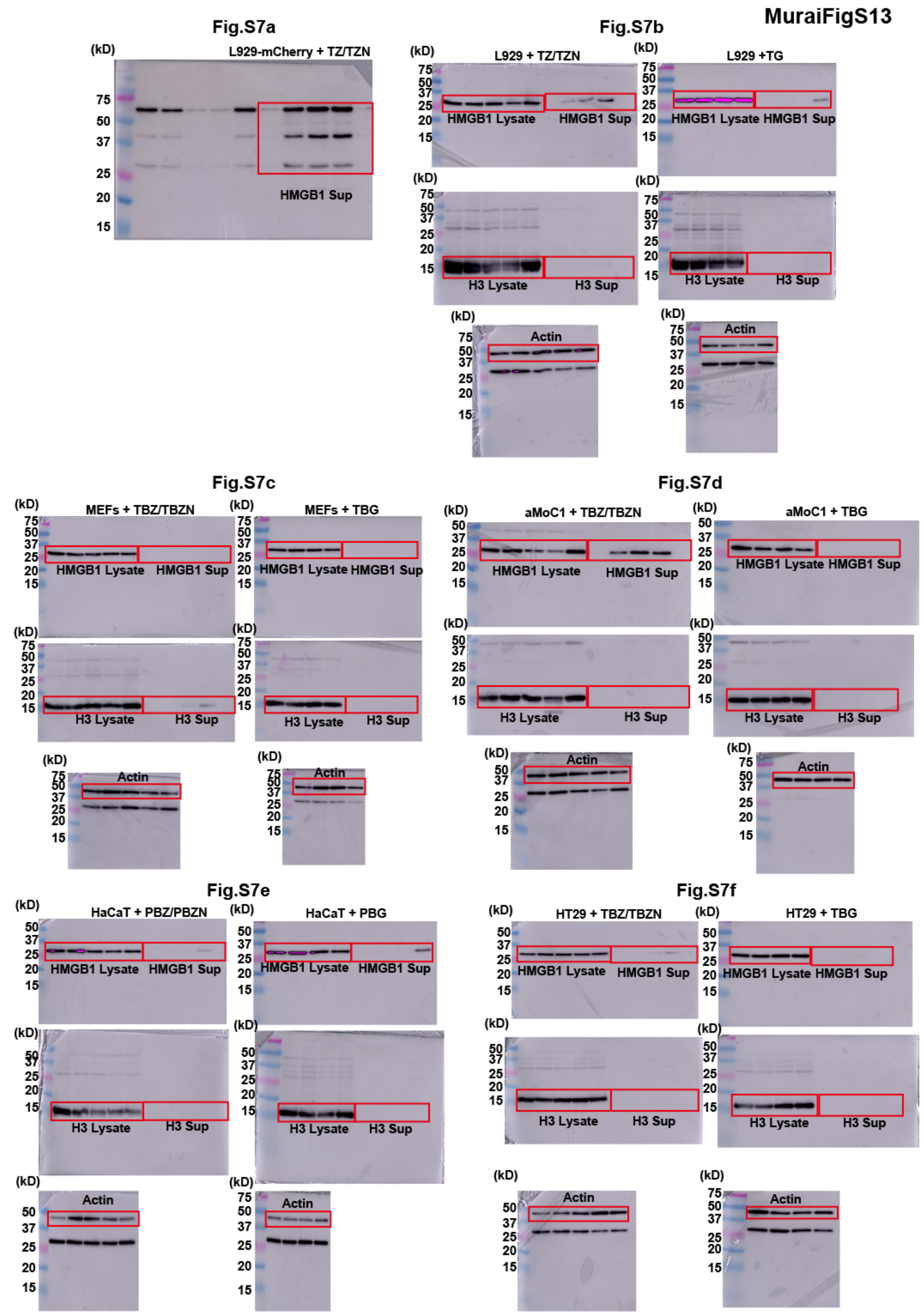

Supplementary Figure 13 | Uncropped blots for Fig. 7.

Supplementary Table 1. Synthesized double-stranded oligonucleotides.

a) Δα3 (*XhoI-HindIII*)

```
CTCGAGCAAGCCGAATCCGTTGGGATTGTACGGTTTACCTTCAACGACGAGATCAAGACGATGA
||||||||||||||||||||||||||||||||||||||||||||||||||||||||||||||||
GAGCTCGTTCGGCTTAGGCAACCCTAACATGCCAAATGGAAGTTGCTGCTCTAGTTCTGCTACT

AGAAGTTTGACAGCCCCAACATTCTGCGCATTTTCGGGATCTGCATAGACCAGACAGTGAAGCC
||||||||||||||||||||||||||||||||||||||||||||||||||||||||||||||||
TCTTCAAACTGTCGGGGTTGTAAGACGCGTAAAAGCCCTAGACGTATCTGGTCTGTCACTTCGG

TCCAGAGTTCTCTATCGTCATGGAGTACTGTGAACTGGGCACATTGAGGGAACTTCTGGATCGA
||||||||||||||||||||||||||||||||||||||||||||||||||||||||||||||||
AGGTCTCAAGAGATAGCAGTACCTCATGACACTTGACCCGTGTAACTCCCTTGAAGACCTAGCT

GAGAAGGATCTGACTATGAGTGCAGGCGGCTCTGCTGGTGGGAGCGCGGGTGGATCAGCTGGAG
||||||||||||||||||||||||||||||||||||||||||||||||||||||||||||||||
CTCTTCCTAGACTGATACTCACGTCCGCCGAGACGACCACCCTCGCGCCCACCTAGTCGACCTC

GGTCTGCAGGCGGGAGTGCCGGAGGCTCAGCCGGTGGATCCGCCGGTGGAAGCGCTGGCGGCGG
||||||||||||||||||||||||||||||||||||||||||||||||||||||||||||||||
CCAGACGTCCGCCCTCACGGCCTCCGAGTCGGCCACCTAGGCGGCCACCTTCGCGACCGCCGCC

ATATCAGGTGAAGCTT
||||||||||||||||
TATAGTCCACTTCGAA
```

b) Δabc (*XhoI-HindIII*)

```
CTCGAGCAGGCCGAAAGTGTTGGAATCGTACGGTTCACTTTCAATGACGAGATCAAAACCTCAG
||||||||||||||||||||||||||||||||||||||||||||||||||||||||||||||||
GAGCTCGTCCGGCTTTCACAACCTTAGCATGCCAAGTGAAAGTTACTGCTCTAGTTTTGGAGTC

CTGGAGGCAGTGCTGGCGGCAGCGCAGGTGGAAGTGCGGGTGGCAGTGATCAAGCAGGGGGGTC
||||||||||||||||||||||||||||||||||||||||||||||||||||||||||||||||
GACCTCCGTCACGACCGCCGTCGCGTCCACCTTCACGCCCACCGTCACTAGTTCGTCCCCCCAG
```

```
CGCTGGGGGCTCCGCTGGCGGGTCGGCCGGTGGAAGTGCAGGCCTGAGGGAGCTCCTGGATAGA
||||||||||||||||||||||||||||||||||||||||||||||||||||||||||||||||
GCGACCCCCGAGGCGACCGCCCAGCCGGCCACCTTCACGTCCGGACTCCCTCGAGGACCTATCT

GCAGGAGGCAGCGCAGGTGGTGTGCGCAGCCTCCTAGTCCTGAGGGCAGCCAGAGGCTTATACG
||||||||||||||||||||||||||||||||||||||||||||||||||||||||||||||||
CGTCCTCCGTCGCGTCCACCACACGCGTCGGAGGATCAGGACTCCCGTCGGTCTCCGAATATGC

GTGGAAGCGCTGGTGGATCAGCCGGCGGAAGCGCTGGCGGCTCCGCTGGAGGATCAGCCGGAGG
||||||||||||||||||||||||||||||||||||||||||||||||||||||||||||||||
CACCTTCGCGACCACCTAGTCGGCCGCCTTCGCGACCGCCGAGGCGACCTCCTAGTCGGCCTCC

CTCCGCAGGAAAGCTT
||||||||||||||||
GAGGCGTCCTTTCGA
```

c) hSMART (*XhoI-StuI*)

```
CTCGAGCAGGCTGGCAGCATTGCAATAGTGAGGCAGACTTTCAATAAGGAGATCAAAACCATGA
||||||||||||||||||||||||||||||||||||||||||||||||||||||||||||||||
GAGCTCGTCCGACCGTCGTAACGTTATCACTCCGTCTGAAAGTTATTCCTCTAGTTTTGGTACT

AGAAATTCGGTACCAGTGCTGGTGGTAGTGCTGGTGGTAGTGCTGGTGGTAGTGCTGGTGGTAG
||||||||||||||||||||||||||||||||||||||||||||||||||||||||||||||||
TCTTTAAGCCATGGTCACGACCACCATCACGACCACCATCACGACCACCATCACGACCACCATC

TGCTGGTGGTTCCGGCAGTGCTGGTGGTAGTGCTGGTGGTACCCTGAGGGAGCTGTTGGATAGG
||||||||||||||||||||||||||||||||||||||||||||||||||||||||||||||||
ACGACCACCAAGGCCGTCACGACCACCATCACGACCACCATGGGACTCCCTCGACAACCTATCC

GAAAGTGCTGGTGGTCTTGGCAAGCGCATGGTCCTAGTCCTGGGGGCAGCCCGAGGCCT
|||||||||||||||||||||||||||||||||||||||||||||||||||||||||||
CTTTCACGACCACCAGAACCGTTCGCGTACCAGGATCAGGACCCCCGTCGGGCTCCGGA
```
