# Supplementary material for: A FRET biosensor for necroptosis uncovers two different modes of the release of DAMPs
Source: Nat Commun. 2018 Oct 26;9:4457. doi: 10.1038/s41467-018-06985-6 (PMC6203740; doi:10.1038/s41467-018-06985-6)
Supplement: Supplementary file 3 — Description of Additional Supplementary Files [file 41467_2018_6985_MOESM3_ESM.pdf]

Description of Additional Supplementary Files

File Name: **Supplementary Movie 1**
Description: **Live imaging of necroptosis in L929 cells**. SMART live imaging of TNF + zVAD (TZ)-stimulated L929 cells (left), and merged images of SYTOX (cyan) and DIC (right). Scale bar, 20 μm.

File Name: **Supplementary Movie 2**
Description: **Inhibition of necroptosis in L929 cells.** SMART live imaging of TNF + zVAD + GSK'872-stimulated L929 cells (left), and merged images of SYTOX (cyan) and DIC (right). Scale bar, 20 μm.

File Name: **Supplementary Movie 3**
Description: **Live imaging of apoptosis in L929 cells.** SMART live imaging of TNF + GSK'872-stimulated L929 cells (left), and merged images of SYTOX (cyan) and DIC (right). Scale bar, 20 μm.

File Name: **Supplementary Movie 4**
Description: **Live imaging of necrosis in L929 cells.** SMART live imaging of CCCP-stimulated L929 cells (left), and merged images of SYTOX (cyan) and DIC (right). Scale bar, 20 μm.

File Name: **Supplementary Movie 5**
Description: **Live imaging of necroptosis in MEFs.** SMART live imaging of TBZ-stimulated MEFs (left), and merged images of SYTOX (cyan) and DIC (right). Scale bar, 20 μm.

File Name: **Supplementary Movie 6**
Description: **Live imaging of necroptosis in aMoC1 cells.** SMART live imaging of TBZ-stimulated aMoC1 cells (left), and merged images of SYTOX (cyan) and DIC (right). Scale bar, 20 μm.

File Name: **Supplementary Movie 7**
Description: **Live imaging of extracellular release of HMGB1 during necroptosis**. SMART live imaging of TZ-stimulated L929 cells expressing HMGB1-mCherry (left), merged images of nuclear HMGB1-mCherry and DIC (middle), and imaging of HMGB1-mCherry (right). Scale bar, 20 μm.

File Name: **Supplementary Movie 8**
Description: **Live imaging of HMGB1 release during necroptosis.** SMART live imaging of TZ-stimulated L929 cells expressing HMGB1-mCherry (upper), merged images of intracellular HMGB1-mCherry and BF (middle), and LCI-S of HMGB1-mCherry (lower). Scale bar, 50 μm

File Name: **Supplementary Movie 9**
Description: **Live imaging of a burst-mode of HMGB1 release during necroptosis.** LCI-S of TZ-stimulated L929 cells expressing HMGB1-mCherry (left), and merged images of intracellular HMGB1-mCherry and BF (right). Scale bar, 25 μm.

File Name: **Supplementary Movie 10**
Description: **Live imaging of a sustained-mode of HMGB1 release during necroptosis.** LCI-S of TZ-stimulated L929 cells expressing HMGB1-mCherry (left), and merged images of intracellular HMGB1-mCherry and BF (right). Scale bar, 25 μm.
